# Identifying strategies for sustaining evidence-based HIV prevention interventions for AYA in Nigeria: evidence from a crowdsourcing open call

**DOI:** 10.3389/fpubh.2026.1853507

**Published:** 2026-07-29

**Authors:** Juliet Iwelunmor, Titilola Gbaja-Biamila, Folahanmi T. Akinsolu, Olunike R. Abodunrin, Chiyere Arinze, Jane Okwuzu, Temitope Ojo, Olufunto Olusanya, Peter Kalulu, Ashley Bradon, Ucheoma Nwaozuru, Suzanne Day, Joseph D. Tucker, Oliver C. Ezechi

**Affiliations:** 1 Division of Infectious Diseases, Washington University School of Medicine in St. Louis, St. Louis, MO, United States; 2 Clinical Sciences Department, Nigerian Institute of Medical Research, Lagos, Nigeria; 3 Center for Reproduction and Population Health Studies, Nigerian Institute of Medical Research, Lagos, Nigeria; 4 Department of Public Health, Lead City University, Oyo, Nigeria; 5 Department of Biostatistics and Epidemiology, Nanjing Medical University, Jiangsu, China; 6 Department of Implementation Science, Division of Public Health Sciences, Wake Forest School of Medicine, Winston-Salem, NC, United States; 7 Department of Medicine, University of North Carolina at Chapel Hill, Chapel Hill, NC, United States; 8 Faculty of Infectious and Tropical Diseases, London School of Hygiene and Tropical Medicine, London, United Kingdom

**Keywords:** adolescents and young adults, HIV prevention, implementation science, Nigeria, sustainability

## Abstract

**Introduction:**

Sustaining evidence-based HIV prevention interventions remains a persistent challenge, particularly in low- and middle-income countries where programs often rely on time-limited funding. This study explored youth-generated strategies for sustaining HIV prevention interventions among adolescents and young adults (AYA) in Nigeria following initial implementation.

**Methods:**

We conducted a national crowdsourcing open call inviting Nigerian AYA (aged 14–24 years) to propose strategies for sustaining HIV prevention services through maintaining intervention activities (A), preserving benefits (B), and strengthening capacity (C). The open call was part of the 4 Youth By Youth (4YBY) initiative. Submissions were received as text, images, or short videos and were de-identified prior to analysis. Two trained qualitative researchers conducted inductive thematic analysis with iterative codebook development. Emergent findings were subsequently interpreted using the PLAN (People, Learning, Adaptation, and Nurturing) framework and the Dynamic Sustainability Framework to examine youth-defined sustainment mechanisms. Demographic data were analyzed descriptively.

**Results:**

Of 105 submissions received, 70 met eligibility criteria and were included in the analysis. Youth proposed actionable strategies across three sustainment domains: maintaining delivery activities, preserving intervention value, and strengthening enabling infrastructure. Strategies included youth leadership development, institutional integration, digital adaptation, stigma reduction, participatory monitoring, and diversified financing. Across domains, youth emphasized relational engagement, adaptive learning, and capacity building as mechanisms for long-term viability.

**Conclusion:**

Nigerian AYA articulated practical, context-responsive strategies for sustaining HIV prevention interventions. Centering youth perspectives highlights relational, structural, and adaptive mechanisms necessary for sustaining evidence-based interventions in resource-limited settings.

## Introduction

Sustainment is increasingly regarded as a foundational goal for global health research and implementation science. In Africa, where health systems are often fragile and donor-dependent, failing to sustain evidence-based interventions can lead to the erosion of critical public health gains (1–3). Sustainment has been defined as the extent to which an evidence-based intervention continues to be delivered and maintains its intended benefits following initial implementation support (4–6). As a key implementation outcome, sustainment reflects the long-term integration and continued effectiveness of interventions within routine practice and community settings (5). Consequently, implementation science now emphasizes the need to embed sustainment planning early in the intervention lifecycle, viewing sustainment not as a static endpoint but as a dynamic and iterative process influenced by evolving contexts (7–9).

Despite significant investment in HIV prevention programs, sustainment also remains a major challenge. Implemented programs often falter due to lack of integration into health systems, weak community ownership, and limited mechanisms for long-term viability (2, 10). This challenge is particularly acute for interventions focused on adolescents and young adults (AYA), who not only represent a high-risk population, accounting for nearly one-third of all new HIV infections globally (11, 12), but are also often excluded from the implementation and/or sustainment of the very programs designed to serve them.

Nigeria offers a compelling context for advancing the sustainment of evidence-based HIV prevention interventions. With an estimated 60% of its population under age 25 (13), the country is home to one of the largest AYA populations in Africa. It also bears a high HIV burden, with adolescents and young adults (ages 15–24 years old) accounting for nearly half of all new infections in the country (11, 14). These demographic and epidemiological dynamics highlight the urgent need for AYA-focused strategies that can be used to sustain evidence-based HIV prevention interventions over time. Moreover, Nigeria’s diverse socio-cultural and policy landscape provides an ideal setting to explore scalable, AYA-led models for sustainment that can inform efforts across the region.

Emerging evidence shows that AYA-led strategies, where young people are engaged as co-designers, implementers, and evaluators, lead to higher levels of acceptability, engagement, and sustained outcomes (15–17). Therefore, understanding how AYA conceptualize and envision sustainment is not just beneficial, it is essential for ensuring the long-term impact of HIV prevention interventions. While many HIV prevention programs target AYA, few studies explore how young people themselves conceptualize the sustainment of evidence-based interventions. Existing models often overlook AYA voices or their potential as partners with generating solutions that can be used to sustain these interventions (18–20). This study addresses that gap by analyzing AYA-generated strategies for sustaining evidence-based HIV prevention interventions within community and AYA-centered delivery contexts. The goal of this study is to identify and analyze AYA-generated sustainment strategies for HIV prevention interventions in Nigeria. Findings may inform efforts to strengthen AYA-engaged sustainability planning for evidence-based HIV prevention programs across similar sub-Saharan African settings.

## Methods

### Crowdsourcing open call design

Crowdsourcing open calls are participatory research approaches that solicit ideas or solutions from communities in response to a structured prompt, with submissions evaluated using predefined criteria (17, 21, 22). This method has been increasingly applied in global health to identify innovative, community-driven strategies for addressing complex public health challenges, particularly among AYA populations (17, 21, 22). Our team has previously used crowdsourcing approaches to generate AYA-driven HIV prevention solutions in Nigeria (23, 24).

This study was part of a national AYA-led crowdsourcing open call to identify creative, AYA-driven strategies to sustain the 4 Youth by Youth (4YBY) HIV prevention interventions in Nigeria (4, 17, 21, 22). 4YBY is an AYA-centered initiative that leverages crowdsourcing, peer networks, and human-centered design to expand access to HIV self-testing and prevention services (21, 22). The open call aimed to generate AYA-authored ideas to sustain 4YBY services while nurturing existing relationships, practices, procedures, and systems in community settings (22).

### Recruitment and data collection

Submissions to the open call were accepted between 26 October and 27 November 2022. Participants were invited to respond to the following prompt: *“How might we sustain the 4YBY HIV prevention services while nurturing our existing relationships, practices, procedures, and services in our communities?”*

Submissions could be made as written text (up to 500 words), images (up to 5 MB), or short videos (up to 3 min). Entries were submitted via Google Forms, email, or WhatsApp and were accepted in English.

Eligible participants were Nigerian adolescents and young adults aged 14–24. Submissions could be made individually or as part of a team. For team submissions, demographic information was collected only from the primary contact person. There were no restrictions based on geographic location, educational status, or prior affiliation with the 4YBY initiative.

The open call was organized by the Nigerian Institute of Medical Research (NIMR) in partnership with the 4YBY partner institutions. Recruitment for participants of the open call was conducted through a call-for-submissions which was circulated through multiple AYA-facing channels, including the 4YBY website, Facebook, Instagram, WhatsApp networks, and in-person engagement at AYA-serving community hubs. A Youth Advisory Board (YAB) composed of AYA members supported the design, promotion, and review processes to ensure AYA relevance, accessibility, and inclusivity.

### Screening and selection of submissions for analysis

A total of 105 submissions were received. Two members of the research team independently screened all entries for eligibility based on their relevance to the open call prompt. Submissions that did not address the sustainment of HIV prevention interventions were excluded.

Eligible submissions were subsequently reviewed by a multidisciplinary judging panel consisting of HIV researchers, implementation scientists, community organization representatives, AYA advisors, and public health experts. As part of the crowdsourcing contest process, each submission was evaluated using five predefined criteria: (1) clarity, defined as how clearly the sustainment strategy was articulated; (2) desirability, reflecting its perceived relevance and appeal to AYA populations; (3) innovation, referring to the novelty of the proposed approach; (4) feasibility, assessing the practicality of implementation within the Nigerian context; and (5) potential for sustainable impact, reflecting the likelihood that the proposed strategy could contribute to the long-term continuation of HIV prevention services (17, 21). Each criterion was scored on a scale of 1 to 10, with higher scores indicating stronger performance (21).

The judging criteria were adapted from previously published crowdsourcing open call methods and refined collaboratively by the research team and Youth Advisory Board to ensure relevance to AYA-centered HIV prevention and sustainment. Although the primary purpose of scoring was to identify finalists for the crowdsourcing contest, scores were also used to determine whether submissions contained sufficient conceptual detail for qualitative analysis. While all submissions reflected AYA perspectives, some consisted of brief statements with limited explanation, making meaningful interpretation difficult. Therefore, submissions with an average score of ≥6.0 were retained for analysis, representing entries that demonstrated at least moderate clarity, feasibility, innovation, and relevance to sustainment. Scores were not used during coding, theme development, or interpretation of findings.

### Data management

All eligible submissions were compiled into a single dataset and de-identified prior to analysis. Text-based submissions were analyzed directly. Spoken content from video submissions was transcribed verbatim. Image submissions and non-textual visual content were reviewed alongside any accompanying textual descriptions, and relevant contextual information was captured in analytic memos to support interpretation. Evaluation scores were retained for descriptive purposes but were not used during thematic coding.

### Data analysis

All submissions were imported into NVivo 12 software (25) for data management and analysis (24). Two trained qualitative researchers independently reviewed the dataset to familiarize themselves with the content and identify preliminary codes. An initial codebook was developed through iterative discussion, applied to a subset of submissions, and subsequently refined before being applied to the full dataset (26).

An inductive thematic content analysis was conducted to identify recurring patterns and sustainment strategies proposed by participants (27, 28). Following coding, themes were organized within the three predefined sustainment domains (activities, benefits, and capacity) embedded within the open call prompt. These domains served as analytic categories rather than emergent themes. Activities referred to strategies aimed at maintaining intervention delivery and quality over time; benefits captured mechanisms that reinforced the perceived value and responsiveness of the intervention to AYA needs; and capacity encompassed the human, organizational, and technological infrastructures required to support long-term program continuity.

Within each domain, coded data were reviewed to identify recurring sustainment strategies and cross-cutting mechanisms. The PLAN framework (People, Learning, Adaptation, and Nurturing) was subsequently applied as an interpretive lens to examine how AYA-defined sustainment strategies aligned with key sustainment processes (29). PLAN was not used as an *a priori* coding framework; rather, it informed interpretation of the coded data by examining how proposed strategies engaged key actors, supported continuous learning, responded to contextual realities, and nurtured the relational and motivational foundations necessary for sustainment.

Demographic characteristics of participants were analyzed descriptively using frequencies and percentages. While submissions could be made individually or by teams, demographic information was collected only from the primary individual submitting each entry and therefore reflects submitter characteristics rather than all team members.

### Rigor and trustworthiness

Methodological rigor was enhanced through multiple strategies. Coder triangulation was achieved through independent coding and regular analytic discussions to resolve discrepancies and refine interpretations. An audit trail documenting coding decisions and the application of the framework was maintained throughout the analysis.

Verbatim quotes were retained to support interpretive credibility and illustrate analytic themes. The study adhered to the COREQ (Consolidated Criteria for Reporting Qualitative Research) checklist and applied Lincoln and Guba’s criteria of credibility, dependability, confirmability, and transferability to strengthen trustworthiness (30, 31).

### Ethical considerations

Ethical approval was obtained from the Institutional Review Board at the Nigerian Institute of Medical Research, Lagos, Nigeria. Online informed consent was obtained from all participants of the open call prior to submitting their ideas. Participation was voluntary, and no personally identifiable or sensitive data were collected beyond the demographic metadata required for entry validation.

## Results

Of the 105 individuals who submitted ideas to the open call, 52% of submissions were made by men and 48% by women. Participants’ ages ranged from 14 to 24 years old, with the majority being 22 years old. The highest level of education completed was secondary school, completed by 58.7% of participants, and most participants were currently students (84.6%). Prior to submitting an idea to the open call, 77.0% of participants had heard of HIV self-testing and 58.7% of the participants had heard of PrEP. However, only 43.0% were aware of the 4YBY program before entering the contest, suggesting that most submitted ideas were generated without prior direct exposure to the 4YBY program.

### Sustainability-focused analytic framework

Thematic analysis of the 70 AYA-generated submissions was organized around three sustainment domains—activities, benefits, and capacity—reflecting the structure of the open call prompt. These domains served as analytic categories. Activities referred to strategies aimed at maintaining intervention delivery and quality over time, benefits captured mechanisms that reinforced perceived value and responsiveness to AYA needs. Capacity encompassed structural, human, and organizational infrastructures necessary to support long-term program continuity. Within each domain, review of coded data identified cross-cutting mechanisms in sustainment strategies proposed by participants. The PLAN framework (People, Learning, Adaptation, and Nurturing) was subsequently applied as an interpretive lens to examine how AYA-defined strategies reflected key sustainment mechanisms. PLAN dimensions were used to interpret how the proposed sustainment strategies engaged actors, supported adaptive learning, responded to contextual realities, and nurtured relational and motivational foundations for sustainment.

Tables 1–3 presents the sustainment domains and associated cross-cutting sustainment mechanisms.

**Table 1:** AYA-defined sustainment strategies for maintaining intervention activities.

PLAN dimension	Representative sustainment strategies
People	Cultivating AYA leaders and ambassadors to champion ongoing HIV prevention efforts.
Learning	Developing innovative and diversified financing mechanisms to support continued delivery.
Adaptation	Expanding delivery platforms through community embedding, rural outreach, pharmacies, markets, and interactive technologies.
Nurturing	Sustaining team cohesion, partnerships, and periodic engagement forums to reinforce motivation and continuity.

**Table 2:** AYA-defined sustainment strategies for preserving intervention benefits.

PLAN dimension	Representative sustainment strategies
People	Maintaining AYA leadership and peer educator roles to reinforce long-term engagement and empowerment.
Learning	Engaging AYA in participatory monitoring and evaluation to ensure continuous improvement and credibility.
Adaptation	Enhancing service accessibility, including digital platforms and revenue-generating online service extensions.
Nurturing	Sustaining stigma-reduction campaigns and inclusive outreach to reinforce social legitimacy and relevance.

**Table 3:** AYA-Defined sustainment strategies for strengthening implementation capacity.

PLAN dimension	Representative sustainment strategies
People	Institutionalizing AYA fellowships and ambassador programs to build renewable leadership pipelines.
Learning	Training peers to independently design and implement school- and community-based HIV prevention services.
Adaptation	Leveraging digital tools and social media platforms to maintain adaptive, AYA-responsive engagement.
Nurturing	Establishing grassroots, age-appropriate, and culturally relevant training infrastructures that reinforce HIV prevention advocacy across households, schools, and communities.

### Sustaining activities of the 4YBY intervention

AYA-defined strategies for sustaining 4YBY activities clustered around four cross-cutting mechanisms: distributed leadership, financial innovation, adaptive delivery expansion, and relational continuity.

First, distributed AYA leadership emerged as a central sustainment strategy. Rather than positioning AYA solely as beneficiaries, participants consistently proposed ambassador networks, regional AYA leaders, and peer-led extensions of the intervention, particularly in underserved rural settings. These approaches aimed to decentralize ownership and embed leadership capacity within local communities, thereby reducing reliance on centralized project structures.

One participant proposed creating *“a national network of empowered youth leaders advancing HIV prevention through education, advocacy and innovation,”* while another emphasized empowering rural youth as local HIV prevention champions to strengthen community ownership of the intervention.

Second, participants emphasized financial innovation as a learning-based sustainment mechanism. Several proposals suggested diversifying funding streams through partnerships with commercial entities, premium service packaging, and micro-contribution models. These ideas reflected AYA awareness of post-donor vulnerabilities and the need to develop self-sustaining financial pathways. For example, one participant proposed *“applying for grants and exploring partnerships”* while building a financially sustainable youth-driven HIV prevention platform through revenue-generating activities and service innovations.

Third, adaptive expansion of delivery platforms was highlighted as critical for maintaining relevance and reach. AYA proposed integrating 4YBY into pharmacies, markets, community-based organizations, and rural institutions, alongside digital adaptations such as gamified engagement tools. One submission, “Project CESI (Community Engagement Sustainability Innovation),” emphasized embedding HIV prevention services within community-based organizations through trained peer educators, while another proposed adapting intervention materials and delivery channels to better reach rural youth populations. These strategies reflected a recognition that sustained delivery requires responsiveness to shifting social and technological contexts.

Finally, relational continuity and team culture were described as essential for nurturing ongoing implementation. Participants emphasized maintaining partnerships, annual engagement forums, digital tools, and team cohesion to foster belonging, optimism, and resilience among implementers. One submission highlighted the importance of creating *“a resilient and inclusive team culture that fosters collaboration and innovation”* through mentorship, support systems, and active engagement. This human-centered dimension framed sustainment not only as structural continuity but also as the preservation of motivation, trust, and collective identity. Illustrative participant excerpts supporting these strategies are presented in Table 4.

**Table 4:** Illustrative participant excerpts supporting major sustainment strategies.

Sustainment domain	Cross-cutting strategy	Illustrative participant excerpt
Activities	Youth leadership and ownership	*“Project Youth Inclusive aims to create a national network of empowered youth leaders advancing HIV prevention through education, advocacy and innovation.”*
	Financial sustainability	*“Apply for grants and explore partnerships… build a financially sustainable youth-driven HIV prevention programme.”*
	Community integration and adaptation	*“Community-based peer educators should implement the programme through community-based organizations to ensure long-term sustainability.”*
Benefits	Youth empowerment and leadership	*“Young people should be empowered to become leaders, educators and change agents within their communities.”*
	Monitoring, evaluation, and continuous improvement	*“Young monitoring and evaluation officers should track progress and success rates to improve the programme over time.”*
	Stigma reduction and social acceptance	*“Creating a stigma-free society where HIV awareness and prevention are normalized through creative youth-driven initiatives.”*
Capacity	Leadership development and fellowships	*“Establish the 4YBY AIDS Fellowship, an annual training programme for youth ambassadors across different states.”*
	Digital innovation and engagement	*“Establish a dynamic and tech-driven approach to sustain HIV prevention services targeting youth populations.”*

### Sustaining benefits of the 4YBY intervention

AYA-defined strategies within the benefits domain emphasized sustaining the perceived value and legitimacy of the 4YBY intervention over time. Participants framed benefits not only as outcomes, but as mechanisms that justify continued engagement, investment, and ownership.

First, empowerment and leadership development emerged as a central benefit-based sustainment pathway. AYA envisioned themselves as ongoing leaders, educators, and peer advocates, suggesting that strengthening AYA agency would reinforce the intervention’s long-term relevance. By positioning beneficiaries as future implementers, participants linked personal growth with program continuity. One participant proposed creating a youth-led movement in which young people would serve as “leaders, educators and change agents” capable of promoting HIV prevention within their own communities.

Second, maintaining accessibility and service relevance was described as essential for preserving intervention value. Participants emphasized addressing knowledge gaps, reducing misconceptions, and expanding access to HIV prevention services, particularly for underserved and rural AYA. Several submissions highlighted the need to ensure that HIV prevention services remain available to marginalized populations through youth-friendly delivery channels and community outreach. These strategies reflected an understanding that sustained demand depends on visible, ongoing benefit to users.

Third, participatory monitoring and adaptive refinement were proposed as mechanisms for reinforcing program credibility. AYA suggested involvement in monitoring and evaluation processes to ensure accountability and continuous improvement. One submission, “Partnership for Growth,” proposed engaging young monitoring and evaluation officers to track progress and success rates while using findings to continuously improve intervention delivery. This approach framed learning not only as internal program evaluation, but also as a strategy for sustaining trust and national network expansion.

Fourth, financial and platform adaptations were proposed to enhance long-term benefit retention. Ideas such as integrating online purchasing options for HIV self-testing kits reflected attempts to align intervention benefits with evolving digital behaviors while generating potential revenue streams to support continuity. One participant proposed enhancing the intervention platform with an online store for HIV prevention products while simultaneously strengthening partnerships and grant opportunities to support long-term sustainability.

Finally, participants emphasized stigma reduction and psychosocial well-being as enduring benefits that support sustained engagement. Continued creative campaigns, digital storytelling, and targeted outreach were described as ways to reinforce social acceptance and normalize HIV prevention among AYA populations. One participant described the goal of creating “a stigma-free society where HIV awareness and prevention are normalized through creative youth-driven initiatives.” Collectively, these strategies illustrate how AYA framed benefits as drivers of legitimacy, motivation, and long-term value preservation necessary for sustaining HIV prevention interventions. Representative excerpts demonstrating how participants framed sustained benefits are shown in Table 4.

### Sustaining capacity with 4YBY intervention

AYA-defined strategies within the capacity domain emphasized building durable human, institutional, and technological infrastructures to support long-term implementation. Capacity was framed not as short-term training, but as the development of self-sustaining systems that embed HIV prevention within AYA networks and community structures.

First, institutionalized AYA leadership development emerged as a foundational capacity-building strategy. Participants proposed structured ambassador programs, fellowships, and mentored AYA-led initiatives that would formalize leadership pathways across states. These approaches aimed to create a renewable pipeline of trained AYA implementers capable of independently advancing 4YBY objectives, thereby reducing dependence on centralized program oversight. For example, one participant proposed establishing a “4YBY AIDS Fellowship,” described as an annual training program for youth ambassadors across different states that would promote HIV prevention services and strengthen the long-term sustainability of the intervention. Another submission emphasized supporting autonomous youth-led HIV prevention initiatives that would enable young people to independently design and implement projects aligned with the goals of 4YBY.

Second, peer-led knowledge transfer systems were highlighted as mechanisms for distributed implementation. AYA proposed structured training programs within schools and communities to equip peers with skills to design, deliver, and adapt HIV prevention services. One participant proposed a six-week in-school training strategy aimed at fostering peer-to-peer engagement and strengthening youth participation in HIV prevention activities. By embedding knowledge within local AYA networks, these strategies reinforced sustainability through decentralized expertise.

Third, digital and technological capacity was identified as critical for adaptive sustainment. Participants emphasized building technology-enabled engagement platforms, including social media-based programming, AYA-led podcasts, and online dissemination tools. One submission, “Project Hi-Connect,” proposed establishing a “dynamic and tech-driven approach” that leverages social media, digital content creation, and youth-led online platforms to sustain HIV prevention messaging and engagement. These strategies positioned digital fluency as a structural asset for maintaining relevance, reach, and scalability over time.

Finally, grassroots and community-level training infrastructures were described as essential for nurturing multi-level engagement. Participants suggested capacity-building initiatives at household, school, and community levels to ensure that HIV prevention knowledge is continuously reinforced across social environments. One proposal highlighted the need for grassroots training among community-based groups, while another emphasized practical, age-appropriate, and culturally relevant training that would enable young people to disseminate HIV prevention information within households, schools, and communities. This reflects an understanding that sustainable interventions require alignment across interpersonal and institutional systems.

Collectively, these capacity-oriented strategies illustrate AYA recognition that sustainment depends on building enduring human capital, distributed leadership structures, digital infrastructures, and community-embedded knowledge systems. Selected participant excerpts illustrating capacity-focused sustainment strategies are presented in Table 4.

Across the three sustainment domains, participant submissions consistently emphasized youth leadership, community ownership, adaptive learning, digital innovation, and long-term capacity development as mechanisms for sustaining HIV prevention interventions. While the specific strategies varied across activities, benefits, and capacity domains, these cross-cutting priorities were evident throughout the submissions. Representative excerpts illustrating the major sustainment strategies identified during the analysis are presented in Table 4.

## Discussion

This study identified three interrelated sustainment mechanisms articulated by Nigerian AYA: distributed AYA leadership, adaptive financial and delivery innovation, and the development of enduring human and relational capacity infrastructures. Together, these findings suggest that AYA conceptualize sustainment not as simple continuation of activities, but as a dynamic and evolving process requiring structural embedding, value preservation, and adaptive responsiveness. In LMIC settings where HIV prevention interventions are frequently donor-dependent and resource-constrained (4, 9), such AYA-defined sustainment pathways offer important insights into how long-term program continuity may be achieved (1, 3).

First, distributed leadership emerged as a central sustainment strategy. AYA consistently positioned themselves not merely as beneficiaries of HIV prevention services but as ambassadors, peer educators, and regional coordinators responsible for extending intervention reach. This reframing shift sustainability discourse from institutional maintenance to decentralized ownership. While sustainability frameworks often emphasize organizational integration and resource stability (32), fewer studies have empirically examined how end-users conceptualize their own role in long-term continuity (6, 32). The AYA-generated strategies in this study illustrate that leadership transfer and peer-network diffusion are viewed as foundational mechanisms for sustaining intervention relevance and reach. This finding aligns with equity-oriented implementation science, which underscores the importance of participatory engagement and shared ownership for long-term program viability (33).

Second, AYA emphasized adaptability as essential to sustainment. Rather than prioritizing fidelity preservation alone, participants proposed diversified financing mechanisms, digital delivery platforms, rural embedding, and integration with community-based institutions. These strategies reflect an understanding that interventions must evolve alongside changing social, technological, and economic contexts. This perspective aligns with the Dynamic Sustainability Framework (DSF) (9, 34), which conceptualizes sustainability as the ongoing interaction among interventions, implementation contexts, and broader ecological systems. AYA proposals demonstrated this dynamism through continuous learning, financial innovation, and platform expansion. In doing so, participants reinforced the DSF principle that long-term effectiveness depends not on static replication but on adaptive refinement in response to contextual shifts (32).

Third, relational and motivational infrastructures were identified as critical, yet often underrecognized, components of sustainment. AYA highlighted the importance of team cohesion, annual gatherings, digital engagement forums, stigma-reduction campaigns, and community-level advocacy training. These strategies suggest that sustainment requires nurturing collective identity, trust, and belonging alongside structural continuity. Traditional sustainability models emphasize continued activities and institutional integration (32), but the present findings extend this perspective by foregrounding emotional and relational dimensions as drivers of long-term engagement (32). By framing sustainment as both technical and social, AYA articulated a multidimensional understanding of what enables interventions to endure (1, 29).

Importantly, AYA also linked sustainment to the preservation of intervention benefits. Participants emphasized maintaining service accessibility, reducing stigma, addressing knowledge gaps, and ensuring ongoing responsiveness to AYA needs. This reflects the principle of “fit” between interventions and end-user values, increasingly recognized as central to long-term viability (2, 4, 6). In this framing, continued engagement depends on the ability of interventions to remain relevant, responsive, and beneficial to the populations they serve. By coupling benefit preservation with adaptive learning and participatory monitoring, AYA strategies suggest that legitimacy, accountability, and responsiveness may serve as important mechanisms supporting long-term sustainment.

Although sustainment is commonly defined as the continued delivery of interventions and maintenance of intended benefits over time and is recognized as a key implementation outcome in implementation science (2, 7), this study did not directly measure sustainment as an implementation outcome. Rather, it explored AYA-generated strategies perceived as important for achieving sustainment following initial implementation. Consequently, the findings should be interpreted as youth-defined pathways to sustainment rather than empirical evidence of sustained intervention delivery or effectiveness.

Collectively, these findings extend foundational sustainability frameworks proposed by Shediac-Rizkallah and Bone (6), which conceptualize conceptualize sustainment as a multidimensional process involving leadership transfer, adaptive financing, community integration, and relational engagement, extending traditional sustainability discussions that often focus primarily on organizational or financial continuity. While those domains remain highly relevant, the present analysis illustrates how AYA operationalize these domains through mechanisms of distributed leadership, adaptive learning, and relational nurturing. The application of the PLAN framework in this study provided an interpretive structure to understand how people, learning, adaptation, and nurturing operate across activities, benefits, and capacity domains (4, 6, 17, 32). Rather than introducing a new conceptual model, this analysis demonstrates how existing sustainment frameworks can be operationalized through AYA-defined strategies in real-world contexts.

These findings have relevance for HIV prevention programming among AYAs in LMICs (4, 9, 35, 36). AYA prioritized peer-led campaigns, digital innovation, participatory monitoring, and grassroots training infrastructures that align with their lived experiences and social realities. By embedding sustainment strategies within AYA networks and community institutions, these approaches enhance the likelihood that interventions remain visible, valued, and responsive over time (11, 37, 38). The emphasis on technology-enabled engagement further reflects recognition of evolving communication ecosystems that shape AYA health behaviors.

Moreover, the findings challenge traditional top-down sustainability planning approaches (4, 9, 39). AYA-generated strategies integrated technical (financing, distribution platforms), social (peer networks, community embedding), and emotional (motivation, belonging, stigma reduction) dimensions of sustainment. Such multidimensional strategies illustrate that AYA can articulate sophisticated systems-level mechanisms for maintaining intervention continuity. These findings reinforce calls within implementation science to incorporate community-driven perspectives and relational trust as central components of long-term program success (33).

An additional finding worth noting is that although most participants reported prior awareness of HIV self-testing and more than half were aware of PrEP, fewer than half had previous knowledge of the 4YBY intervention. This suggests that the sustainment strategies identified in this study were not solely generated by existing beneficiaries or individuals closely involved with the program. Instead, participants appeared to draw on broader awareness of HIV prevention approaches and youth health needs when proposing sustainment mechanisms. From a sustainability perspective, this finding is important because it indicates that support for sustaining HIV prevention interventions may extend beyond current program users. The ability of youth with limited direct exposure to 4YBY to generate actionable sustainment strategies may contribute to strengthening community ownership, intervention legitimacy, and long-term continuity by incorporating perspectives from both program participants and the wider youth population. This observation is consistent with sustainability literature highlighting the importance of stakeholder engagement, community support, and broad ownership in promoting the long-term maintenance of public health interventions (3, 5, 6, 32).

The findings have several implications for HIV prevention policy and practice in LMICs. First, institutionalizing AYA participation within governance and implementation structures may strengthen long-term program continuity. Formal ambassador networks, AYA advisory roles, and decentralized peer-leadership models align with participants’ emphasis on distributed ownership (11, 37). Second, adaptive financing and digital innovation should be integrated into sustainability planning from the outset. Diversifying funding streams and leveraging technology-enabled delivery mechanisms may reduce reliance on time-limited donor cycles while enhancing AYA engagement (9, 37, 38). Third, investment in AYA capacity-building is essential for sustainment. Structured training in participatory planning, digital communication, and adaptive implementation can support the gradual transfer of ownership from external implementers to local actors. Innovation hubs, mentorship programs, and community-level training infrastructures offer practical pathways for operationalizing these capacities.

This study has several limitations. First, participation in the crowdsourcing open call was self-selected, potentially overrepresenting AYA with a pre-existing interest in HIV prevention and community engagement. Second, demographic information was limited to characteristics collected during open call registration. Data on religion, HIV status, and detailed geographic distribution were not collected, limiting our ability to examine whether sustainment strategies differed across these potentially important subgroups. Third, findings are context-specific to Nigeria and may not fully reflect sustainment perspectives across other sociocultural settings in sub-Saharan Africa.

In addition, this study explored AYA-generated strategies perceived as important for sustaining HIV prevention interventions rather than directly measuring sustainment as an implementation outcome. Consequently, the findings should be interpreted as proposed pathways to sustainment rather than evidence of sustained intervention delivery or effectiveness. Future research should examine how AYA-generated sustainment strategies perform when implemented longitudinally, assess their adaptability across diverse health system contexts, and evaluate their contribution to actual intervention sustainment over time.

## Conclusion

This study demonstrates that adolescents and young adults possess the conceptual and strategic capacity to plan for the sustainment of evidence-based HIV prevention interventions. Through a national crowdsourcing open call, Nigerian AYA articulated structured, context-responsive sustainment strategies that address delivery continuity, intervention value, and long-term capacity. AYA contributions to sustainment strategies expand prevailing models by foregrounding relational, emotional, and cultural dimensions of sustainment alongside technical and institutional considerations. As LMICs seek to accelerate progress toward ending HIV/AIDS, embedding AYA leadership within sustainability planning will be essential for ensuring durable, equitable, and contextually grounded health interventions.

## Data Availability

The datasets presented in this study can be found in online repositories. The names of the repository/repositories and accession number(s) can be found in the article/supplementary material.
